# Exploring Task-Related EEG for Cross-Subject Early Alzheimer’s Disease Susceptibility Prediction in Middle-Aged Adults Using Multitaper Spectral Analysis

**DOI:** 10.3390/s25010052

**Published:** 2024-12-25

**Authors:** Ziyang Li, Hong Wang, Jianing Song, Jiale Gong

**Affiliations:** 1 Department of Mechanical Engineering and Automation, Northeastern University, Wenhua Street, Shenyang 110819, China; 2 Senzhigaoke Company Limited, Gaoke Street, Shenyang 110002, China

**Keywords:** AD detection, cross-subject, task-state EEG, multitaper, machine learning

## Abstract

The early prediction of Alzheimer’s disease (AD) risk in healthy individuals remains a significant challenge. This study investigates the feasibility of task-state EEG signals for improving detection accuracy. Electroencephalogram (EEG) data were collected from the Multi-Source Interference Task (MSIT) and Sternberg Memory Task (STMT). Time–frequency features were extracted using the Multitaper method, followed by multidimensional reduction techniques. Subspace features (F24 and F216) were selected via *t*-tests and False Discovery Rate (FDR) multiple comparisons correction, and subsequently analyzed in the Time–Frequency Area Average Test (TFAAT) and Prefrontal Beta Time Series Test (PBTST). The experimental results reveal that the MSIT task achieves optimal cross-subject classification performance using the Support Vector Machine (SVM) approach with the TFAAT feature set, yielding a Receiver Operating Characteristic Area Under the Curve (ROC AUC) of 58%. Similarly, the Sternberg Memory Task demonstrates classification ability with the logistic regression model applied to the PBTST feature set, emphasizing the beta band power spectrum in the prefrontal cortex as a potential marker of AD risk. These findings confirm that task-state EEG provides stronger classification potential compared to resting-state EEG, offering valuable insights for advancing early AD prediction research.

## 1. Introduction

Alzheimer’s disease (AD) is a progressive neurodegenerative disorder that typically manifests in older adults but has pathological changes that may begin decades earlier [1]. The early identification of individuals at risk of AD is crucial for timely intervention and preventive strategies. However, predicting AD risk in healthy middle-aged adults poses significant challenges [2]. The absence of clinical symptoms [3,4] in this population makes conventional diagnostic methods, such as neuroimaging [5,6] or cognitive assessments [7], insufficiently sensitive. Furthermore, the high variability in biological markers across individuals introduces additional complexity [8] to cross-subject classification tasks.

Recent advances in electrophysiology offer promising avenues for early AD risk prediction [9,10,11]. Electroencephalography (EEG), a non-invasive and cost-effective tool, has gained attention for its ability to capture real-time neural dynamics [12]. While resting-state EEG has been widely studied [13], task-related EEG has shown potential in revealing subtle differences in brain activation patterns [14] that may be linked to AD susceptibility. Resting-state EEG studies have demonstrated that changes in spectral power, particularly in the beta and theta bands, are associated with cognitive decline [15]. However, resting-state signals can be confounded by intrinsic neural noise and lack the specificity provided by cognitive task engagement. Event-related potential (ERP) studies in individuals at risk for AD may reveal neurophysiologic changes that precede clinical deficits, enabling the early detection and diagnosis of “presymptomatic AD” [16,17]. Most studies on AD risk prediction in healthy populations have focused on preventive interventions [18] or genetic biomarkers [19,20], which are costly and time-consuming [21]. However, neuroelectrophysiology-based studies (e.g., EEG) have not been sufficiently emphasized. How to accurately assess their AD risk in the absence of obvious cognitive symptoms [3] remains an important topic that has not been thoroughly explored.

Task-state EEG introduces controlled cognitive demands, enabling the assessment of brain regions critical for executive functions such as working memory, inhibitory control, and attention. For instance, the Multi-Source Interference Task (MSIT) [22] and Sternberg Memory Task (STMT) [23] are known to activate the prefrontal cortex and related neural circuits. These regions are particularly vulnerable in the early stages of AD. Task-induced neural responses in the beta band, often associated with working memory and attention regulation, may provide sensitive biomarkers for AD risk [24]. However, the high-dimensional, non-stationary nature of EEG data [25] and the inherent inter-individual variability [26] make cross-subject prediction challenging. Therefore, task-state EEG signals deserve further study and validation as potential early predictors.

Multitaper spectral analysis [27] has become a robust method for time–frequency feature extraction in EEG studies [28]. Unlike traditional methods, the Multitaper approach reduces spectral leakage and improves the signal-to-noise ratio, making it particularly suited for analyzing EEG signals with low signal strength [29]. By capturing precise spectral characteristics over short time windows, this method is well equipped to identify task-related neural dynamics associated with AD risk. Coupled with advanced feature selection techniques, such as statistical testing [30] and correction for multiple comparisons [31], Multitaper analysis enables the identification of meaningful subspace features for classification. The application of machine learning in EEG signal analysis has made significant progress in recent years, especially in AD early prediction tasks. Common machine learning methods include logistic regression, Random Forest, SVM, and Gradient Boosting [32]. Each method has its unique advantages for different types of EEG data processing tasks.

In this study, we used several machine learning methods to classify AD risk in a healthy middle-aged population, focusing on evaluating the performance of task-state EEG data under different algorithms. The validity of task-related EEG signals in early AD prediction, as well as the adaptability and predictive power of different methods to data characteristics, are explored through cross-questionnaire validation. This research provides a novel perspective on utilizing task-related EEG and advanced spectral analysis for the cross-subject classification of AD risk. This study aimed to explore the use of task-related EEG signals for the early prediction of AD risk in healthy middle-aged adults. Using the Multitaper method, we extract time–frequency features from EEG data collected during the MSIT and STMT tasks. These features undergo dimensionality reduction and statistical validation to identify robust subspace representations. We conducted two classification experiments: the Time–Frequency Area Average Test (TFAAT) and the Prefrontal Beta Time Series Test (PBTST). The results were expected to demonstrate the superior classification ability of task-state EEG compared to resting-state EEG and highlight the potential of beta band spectral markers as early indicators of AD susceptibility.

## 2. Materials and Methods

### 2.1. Data Preprocessing

The PEARL Neuro database was designed for research on susceptibility to Alzheimer’s disease [33] in middle-aged individuals, where 69 subjects participated in two types of cognitive tasks: MSIT and STMT. It contains genetic data on key risk genes, including apolipoprotein E (APOE) and phosphatidylinositol binding clathrin assembly protein (PICALM), both of which are known to increase the risk of late-onset Alzheimer’s disease. Additionally, the dataset includes resting-state data with eyes open phases, which we used as the control group to explore the potential role of task-related EEG in early Alzheimer’s disease detection. The raw EEG data were recorded using a Brain Products system with 128 electrodes. The online reference electrode is FCz, with a sampling rate of 1000 Hz. Electrode impedances were maintained between 5 and 10 kΩ to ensure high-quality and reliable data collection. In the recording process, only a low-pass filter with a cut-off frequency of 280 Hz was employed, without the application of any notch or high-pass filters. Each participant was required to response 166 MSITs and 144 Sternberg stimulus. Each task included two stimulus scenarios of high/low demand, and the number of trials for each scenario was balanced among the participants. During the execution of the experimental paradigm, the stimulus interval was varied within a specific range to minimize brain adaptation. The participants’ response events were simultaneously recorded. More detailed information about the dataset can be found in its original publication [34].

**Figure 1:**
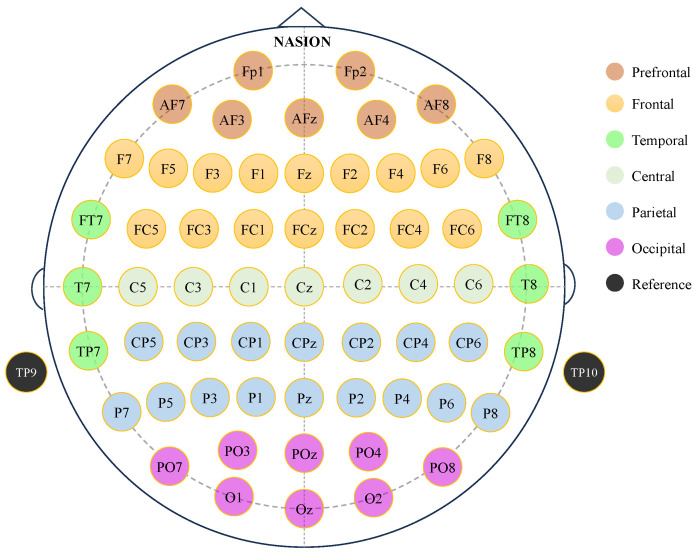
The 60 electrode locations in 10-10 international standard system.

Due to the large amount of raw EEG data provided by the dataset, preprocessing was essential before conducting stimulus-related ERP research. EEG preprocessing followed the principles outlined in Steve J. Luck’s online book [35]. Data preprocessing and experimental analysis were conducted in Matlab (v9.11.0, 2021b) using EEGLAB (v2023.1) [36] and ERPLAB (v12.00) [37]. The EEG signals were re-referenced offline to an approximate average mastoid reference by subtracting the average of TP9 and TP10. Sixty commonly used EEG channels were selected, with their electrode locations categorized into six subregions, as shown in Figure 1: prefrontal, frontal, temporal, central, parietal, and occipital. The data were down-sampled to a frequency of 250 Hz, and a bandpass filter with a half-amplitude cut-off range of 1–30 Hz and a roll-off of 12 dB/octave was applied for denoising. Independent Component Analysis (ICA) and artifact suppression techniques were used to further clean the data. Considering the potential unreliability of γ waves due to the pronounced conduction effects on high-frequency signals in EEG recordings, a half-amplitude cut-off at 30 Hz was applied in this study to filter out γ and higher-frequency waves. For each participant, only trials with correct event responses were included in the analysis. Stimuli with response times shorter than 200 ms were excluded as false triggers. For the MSIT task, trials with response times within 1.5 s after stimulus onset were selected. For the STMT, trials with response times within 2 s after stimulus onset were selected.

For each trial in the MSIT and STMT tasks, the data segment from −80 ms to 1200 ms relative to the stimulus onset was extracted, resulting in a 60-channel by 320-sample-point data matrix. Note that the STMT data segments analyzed in this study were extracted specifically from the retrieval phase of the three working memory phases (Encoding, Maintenance, and Retrieval), with $t_{0}$ representing the moment the cue word was displayed. Epochs were excluded as noisy if the peak-to-peak amplitude in any channel exceeded 150 μV. Certain subjects were excluded from the analysis due to missing event labels, a low proportion of correct response trials, excessive artifacts, or an unacceptable number of bad channels. Finally, the sample size distribution for each subject is shown in Table 1. The MSIT dataset included 62 subjects, and the STMT included 59 subjects. Among them, data from 51 subjects were available for both tasks. The distributions for 51 subjects are representative of those observed in the 62- and 59-subject datasets. Each task was divided into high and low demand levels based on task difficulty. As shown in Table 1, the ratio of available trials for MSIT is higher than that for STMT, and low-demand tasks have a higher ratio of available trials compared to high-demand tasks. The correct response rate of each participant reflects both the demand level and individual executive function, influencing the final number of valid trials. In drawing from the data preprocessing phase, it can be inferred that the signal quality for each participant remains consistent throughout the experiment. Consequently, task difficulty emerges as the primary factor affecting the outcomes. Additionally, resting-state (eyes open) EEG data was segmented into 1.2-s intervals by inserting periodic event labels as virtual stimulus zero points, followed by the same preprocessing steps. The quality of resting-state data is generally higher than that of task-state data, resulting in a larger overall sample size compared to the tasks. However, whether within or across participants, the sample sizes for each class were imbalanced. Ultimately, we categorized the samples into three groups: MSIT, STMT, and Resting-state (Rest). Based on task demand levels, the task-type groups were further subdivided into four subclasses: MSIT-Low (ML), MSIT-High (MH), STMT-Low (SL), and STMT-High (SH).

The ground truth labels for neutral and risky subjects are determined according to the criteria defined in the thesis [38], no risk gene as neutral (null hypothesis, negative), and having a single risk gene as risky (positive). PICALM genes: AA, AG regarded as neutral, GG as risky; APOE genes: ϵ3 as neutral, ϵ4 as risky. Given that aging is the most important factor in AD, it should be matched between and within groups. Differences between the mean and variance of the age of healthy control subjects and patients should be minimized to reduce the dependence of the results on the age factor [39]. So, it is import to ensure sample sizes in the AD risk and neutral groups to avoid bias by gender, age, and other factors. Finally, samples were divided into two groups: neutral non-carriers and risky single-risk individuals. As shown in Table 2, the number of subjects, age distribution, and sex ratio of the two groups did not differ significantly.

### 2.2. Multitaper Time–Frequency Representation

The Multitaper method is a technique used to estimate the spectrum of a signal by applying multiple window functions. This method offers the advantage of providing smoother and more reliable spectral estimates, especially when the signal-to-noise ratio is low. Slepian sequences (also known as Discrete Prolate Spheroidal Sequences, DPSSs) [40] are used as the tapers to ensure that the spectral energy is concentrated within a narrow frequency band, thereby reducing spectral leakage. The Slepian sequences are the solutions to an optimization problem that aims to maximize the concentration of a signal’s energy in a specific frequency band. Mathematically, the tapers are the eigenfunctions of a particular operator designed to maximize spectral concentration. The Slepian sequences $\psi_{k}\left(t\right)$ are obtained as eigenfunctions from the eigenvalue problem, as shown in Equation (1). The spectral estimate for each taper is calculated using Equation (2), and the final spectrum is obtained by averaging across tapers, as described in Equation (3).

(1) $$L\psi_{k}\left(t\right)=\lambda_{k}\psi_{k}\left(t\right)$$

where L is the time–frequency concentration operator, and $\lambda_{k}$ denotes the eigenvalues that determine the concentration of energy in the frequency domain.

(2) $${\hat{S}}_{k}\left(f\right)={\left|\frac{1}{T}\int_{-T/2}^{T/2}x\left(t\right)\psi_{k}\left(t\right)e^{-2\pi ift}dt\right|}^{2}$$

where x(t) is the signal; $\psi_{k}\left(t\right)$ is the *k*-th taper (Slepian sequence); *T* is the duration of the signal; $e^{-2\pi ift}$ represents the Fourier transform; *f* is the frequency.

(3) $$\hat{S}\left(f\right)=\frac{1}{K}\sum_{k=1}^{K}{\hat{S}}_{k}\left(f\right)$$

where ${\hat{S}}_{k}\left(f\right)$ is the spectral estimate from the *k*-th taper; *K* is the number of tapers used; *f* is the frequency variable.

To perform spectral analysis, we define several parameters for the Multitaper method. First, we set the window width (NW = 4), which controls the trade-off between frequency and time resolution. We choose a total of K = 7 tapers to provide a robust spectral estimate. Each taper window has a length of nperseg = 64, and we use nfft = 512 FFT points to ensure high frequency resolution, with the number of FFT points being at least equal to or greater than the window length, and ideally a power of 2 to improve computational efficiency. The overlap between consecutive windows is set to half the window length (noverlap = 32) to reduce spectral leakage. Based on the above settings, the output time–frequency representation for each trial, with 320 data points, will have the dimensions of 60 channels × 512 frequency points × 9 time intervals.

Additionally, the frequency bands of interest are divided into the following ranges: Delta (1–4 Hz), Theta (4–8 Hz), Alpha (8–13 Hz), and Beta (13–30 Hz). These are commonly used to capture different cognitive and neural states. We then perform time–frequency–space dimensionality reduction on the feature space. The power spectrum is averaged over each frequency band, with a frequency resolution of $f_{s}/n_{\mathrm{fft}}$, reducing the 512 frequency points to 4 bands. Spatial dimensionality reduction is achieved by averaging across six brain regions (60 channels), resulting in a dimensionality of 6 × 4 × 9 (referred to as F216). Temporal dimensionality reduction is performed by averaging over the time intervals, leading to a dimensionality of 6 × 4 (referred to as F24). Finally, a *t*-test-based feature screening is applied to the feature space (F216 and F24) with a significance threshold of $10^{-3}$, ensuring greater credibility of the results under more rigorous standards. Here, the results of the *t*-test were corrected for multiple comparisons using the False Discovery Rate (FDR) method.

### 2.3. LPSO Cross-Subject Validation

In this study, we adopted the Leave-p%-Subjects-Out (LPSO) [41] cross-validation method to evaluate the generalizability and robustness of our models. The LPSO approach addresses cross-subject variability by iteratively excluding a certain percentage of subjects (p%) from the training data to serve as the test set, while the remaining subjects are used to train the model. This method provides a flexible and reliable way to assess model performance, ensuring generalizability across diverse subjects. It is particularly valuable for datasets where subject-specific variability can impact performance, requiring a more generalized evaluation. Here, the percentage p is set to 20–25, ensuring that the test set includes data from multiple subjects, thereby increasing the sample size for each validation iteration. To enhance the reliability of the results, we conducted 100 iterations of the experiment, using a series of random seeds (SEED: 42–141) to ensure reproducibility. In each iteration, five neutral subjects and four risky subjects were randomly selected as the test set. Care was taken to maintain identical class distributions between the training and validation sets during each trial. Class ratios were used to weight the evaluation metrics, ensuring fair performance assessment across all categories. This strategy aimed to reduce potential biases and enhance the generalizability of the model’s findings.

This study employed four machine learning methods to explore the predictive capabilities for AD: logistic regression, Random Forest, Support Vector Machine (SVM), and Gradient Boosting. These models were selected for their distinct strengths in classification tasks and their ability to handle complex patterns in the data. Logistic regression is a linear model that provides a clear probabilistic interpretation, making it suitable for understanding relationships between predictors and the outcome. Random Forest is an ensemble method that leverages multiple decision trees to improve prediction accuracy and robustness, reducing overfitting. SVM is a powerful classifier that finds the optimal hyperplane to separate different classes, particularly effective in high-dimensional feature spaces. Gradient Boosting is an ensemble technique that builds strong predictive models by iteratively correcting errors made by previous models, providing high accuracy even with complex datasets. These methods were applied to compare and assess their effectiveness in predicting AD outcomes based on various features extracted from EEG.

### 2.4. Evaluation Metrics

For a more reliable evaluation, we collected 100 trials of the five metrics and the confusion matrix. Additionally, hypothesis testing was performed for significance analysis. The five metrics were accuracy, precision, recall, F1 Score, and ROC AUC.

Accuracy measures the proportion of correctly classified samples among all samples, as shown in Equation (4). However, its effectiveness is limited in imbalanced datasets, where the majority class dominates predictions.

(4) $$\mathrm{Accuracy}=\frac{\mathrm{TP}+\mathrm{TN}}{\mathrm{TP}+\mathrm{FP}+\mathrm{TN}+\mathrm{FN}}$$

where TP: True Positive; TN: True Negative; FP: False Positive; FN: False Negative.

Precision assesses the model’s ability to correctly identify positive instances among all predicted positive instances. For imbalanced datasets, weighted or macro-averaged precision scores (Equation (5)) account for class distribution, ensuring fair evaluation.

(5) $$\mathrm{Weighted}\;\mathrm{Precision}=\sum_{c=1}^{C}w_{c}\cdot \frac{{\mathrm{TP}}_{c}}{{\mathrm{TP}}_{c}+{\mathrm{FP}}_{c}}$$

where *C*: number of classes; $w_{c}=\frac{N_{c}}{\sum_{c=1}^{C}N_{c}}$: the weight for each class is proportional to its sample size $N_{c}$.

Recall, also referred to as sensitivity, measures the model’s ability to identify all actual positive instances. Weighted recall (Equation (6)) is used in imbalanced datasets to ensure minority classes are adequately represented.

(6) $$\mathrm{Weighted}\;\mathrm{Recall}=\sum_{c=1}^{C}w_{c}\cdot \frac{{\mathrm{TP}}_{c}}{{\mathrm{TP}}_{c}+{\mathrm{FN}}_{c}}$$

The F1 Score balances precision and recall, providing a comprehensive evaluation metric. For imbalanced datasets, weighted or macro-averaged F1 Scores (Equation (7)) are typically preferred.

(7) $$\mathrm{Weighted}\;F1=\sum_{c=1}^{C}w_{c}\cdot \frac{2\cdot {\mathrm{Precision}}_{c}\cdot {\mathrm{Recall}}_{c}}{{\mathrm{Precision}}_{c}+{\mathrm{Recall}}_{c}}$$

The ROC AUC (Equation (8)) offers a threshold-independent evaluation of the model’s discrimination capability. With the use of the trapezoidal rule (Equation (9)), AUC scores are numerically approximated. Weighted AUC scores (Equation (10)) are recommended for imbalanced datasets to ensure balanced contributions across all classes.

(8) $$\begin{array}{c} \mathrm{TPR}=\frac{\mathrm{TP}}{\mathrm{TP}+\mathrm{FN}},\;\mathrm{FPR}=\frac{\mathrm{FP}}{\mathrm{FP}+\mathrm{TN}} \end{array}$$

(9) $$\begin{array}{c} {\mathrm{AUC}}_{c}=\sum_{i=1}^{n-1}\left({\mathrm{FPR}}_{i+1}-{\mathrm{FPR}}_{i}\right)\cdot \frac{{\mathrm{TPR}}_{i}+{\mathrm{TPR}}_{i+1}}{2} \end{array}$$

(10) $$\begin{array}{c} \mathrm{Weighted}\;\mathrm{ROC}\;\mathrm{AUC}=\sum_{c=1}^{C}w_{c}\cdot {\mathrm{AUC}}_{c} \end{array}$$

where $\left(FPR_{i},TPR_{i}\right)$ is a point on the ROC curve.

The confusion matrix provides detailed insight into the model’s performance by showing the distribution of correct and incorrect predictions for all classes. In imbalanced datasets, it reveals the model’s bias toward the majority class. Analyzing rows and columns of the confusion matrix can help identify misclassification patterns for minority classes. This matrix, defined in Equation (11), is a tabular representation of predictions versus true labels, providing valuable information for designing strategies like reweighting or resampling.

(11) $$\left[\begin{array}{cc} \mathrm{TP} & \mathrm{FP}\\ \mathrm{FN} & \mathrm{TN} \end{array}\right]$$

## 3. Results

### 3.1. Extracted Time–Frequency Feature Space

In Section 2.2, we obtained the power density spectral features of the EEG signals using multitaper time–frequency analysis and subsequently reduced dimensionality to create two feature spaces: F216 and F24. Rigorous *t*-tests combined with FDR correction for multiple comparisons were applied to the neutral and risk groups in these two feature spaces, screening key features with a significance threshold of $10^{-3}$. Table 3 presents the feature subspaces derived from F24 for seven task conditions, including the ratio of features from the prefrontal and frontal lobes to the total number of features (Total). Except for the rest condition, these ratios consistently exceed 50%, suggesting increased demand on prefrontal and frontal regions during tasks compared to resting state. Such differences likely arise from inter-individual variability, including a personalized compensatory mechanism.

In contrast, the dimensionality of the F216 subspace is much larger, as summarized in Table 4. Resting-state EEG exhibits the highest total feature count but the lowest contribution from the Prefrontal and Frontal lobes, with ratios under 15%. While the ML and MH tasks achieve ratios above 50%, the overall MSIT task ratio remains below 45%. Interestingly, both Table 3 and Table 4 reveal that the STMT task consistently shows higher ratios—Ratio_SL > Ratio_ML, Ratio_SH > Ratio_MH, and Ratio_S > Ratio_M—except in F24, where Ratio_S < Ratio_M due to fewer total features. Notably, in F216, Ratio_SH reaches the highest ratio of 87.1%, with 27 out of 31 features from the prefrontal and frontal regions. This indicates a strong correlation between the activation of the prefrontal and frontal regions during the STMT task and the prediction of AD risk. To validate this finding, we selected the Prefrontal_β_1–9 features from the F216 feature subspace, resulting in a feature dimensionality of samples (×9) to compare classification performance across multiple task conditions. This selection was based on the observation that these nine features within the Prefrontal_β band in the F216 feature space generally meet the significance threshold of $p<1\times 10^{-3}$ under seven task conditions. The only exception is the Prefrontal_β_1 feature from the first temporal segment of the ML condition, where the *p*-value exceeds $1\times 10^{-3}$, but remains below $1\times 10^{-2}$. Therefore, it was included in this analysis. The primary aim of this comparison is to assess the effectiveness of regional features in the context of the STMT task within the same feature space.

As a control, resting-state EEG rarely involves features from the prefrontal and frontal regions, highlighting the significant task-induced activation in these areas. Consequently, task-state EEG features can provide valuable insights into longitudinal changes across subjects or within the same subject over time. Additionally, Table 3 and Table 4 suggest a hidden trend: the number of significant features increases with task demand levels (Total_iH > Total_iL and (Pre&F)_iH > (Pre&F)_iL), except for one special case in F216 (Total_SH < Total_SL). This indicates nuanced differences in brain responses to varying task demands. To explore this further, we conducted cross-subject AD risk tests on EEG data segmented by task demand levels. To simplify the description, we refer to the detection test based on the F24 sub-feature space as the Time–Frequency Area Average Test (TFAAT), and the detection test based on the PFC_β_1–9 features is referred to as the PFC Beta Time Series Test (PBTST). The results of the experiment are presented below.

### 3.2. Performance Comparison

**Figure 2:**
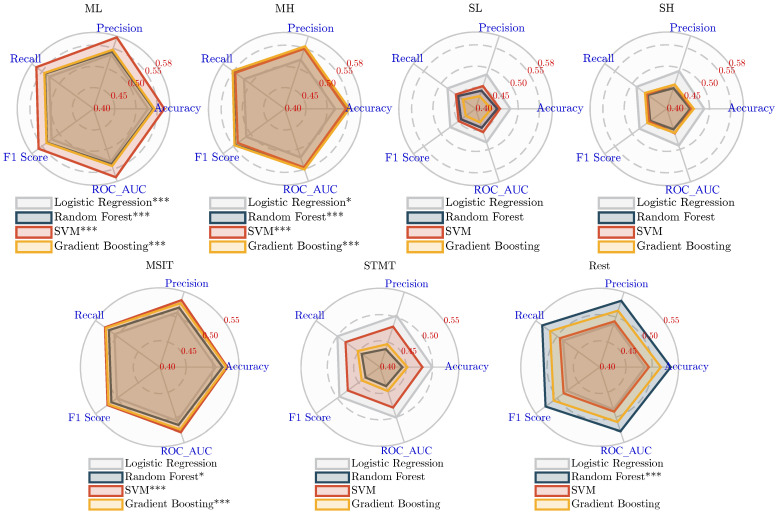
Comparing the performance metrics of the TFAAT across different classification methods with significant levels: * $p<5\times 10^{-2}$, *** $p<1\times 10^{-3}$.

**Figure 3:**
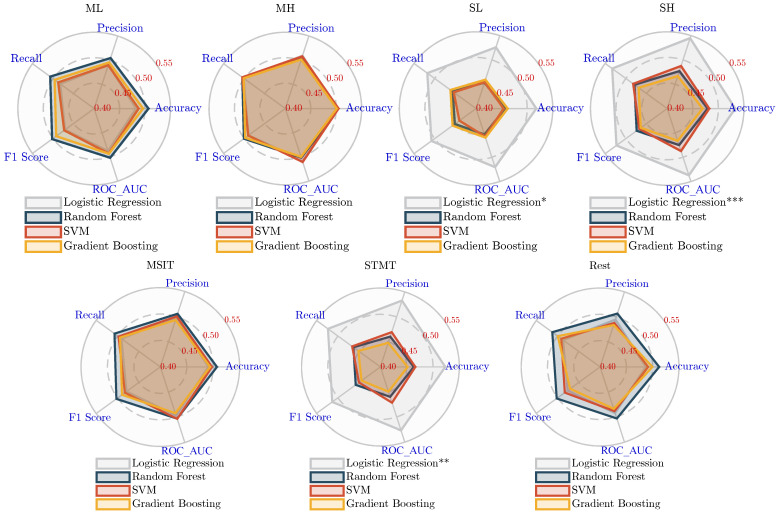
Comparing the performance metrics of the PBTST across different classification methods with significant levels: * $p<5\times 10^{-2}$, ** $p<1\times 10^{-2}$, *** $p<1\times 10^{-3}$.

The five classification performance metrics of the experimental results are presented as radar charts. Each pentagon represents a machine learning method: logistic regression, Random Forest, SVM, and Gradient Boosting. The vertices of the pentagon correspond to the five metrics: accuracy, precision, recall, F1 Score, and ROC AUC. Figure 2 displays the results of the TFAAT, while Figure 3 presents the results of the PBTST. The significance of the results determined by one-tailed *t*-tests ($metric_{i}>0.5$) is indicated as follows: * denotes $p<5\times 10^{-2}$, ** denotes $p<1\times 10^{-2}$, and *** denotes $p<1\times 10^{-3}$.

In the results of the TFAAT experiments, the MSIT task demonstrated the best classification performance compared to the STMT and Rest tasks, as evidenced by the area and significance levels on the radar plot. Specifically, the classification significance ranked in descending order as ML > MH > MSIT. The ROC AUC value of the SVM method under the ML condition reached the maximum value of 0.58, with all four classification methods exhibiting significant differences. Conversely, the STMT task, including the subsets SL and SH, did not show classification significance across any methods. As a control, the Rest condition demonstrated classification significance only with the Random Forest method, achieving a ROC AUC value of 0.53, indicating a potential for classification ability.

Combined with the analysis in Table 3, it can be observed that the ML condition has the fewest features, with only five, yet it demonstrates better classification performance compared to other conditions. While the MH condition includes a greater number of features, its classification effectiveness is notably lower, similar to what is observed with the STMT condition. Despite higher feature dimensions, SL, SH, and STMT do not exhibit observable classification potential. As a control, the Rest condition extracted 17 features but displayed classification ability only with one method. Overall, considering the combined evaluation metrics and feature dimensions, the ML, MH, and MSIT demonstrate outstanding classification performance, achieving significant results across all four methods while utilizing a relatively smaller feature space.

In the PBTST experiment, categorical significance was observed for the Sternberg memory task only in the logistic regression method, as shown in Figure 3. Specifically, the classification performance was ranked from highest to lowest: SH > STMT > SL. The SH condition achieved the highest classification performance, with a ROC AUC value of 0.54. In comparison, the other three methods performed poorly, suggesting that the feature space is more linearly separable. The remaining seven conditions showed little difference in classification performance across all four methods, with none demonstrating classification ability. In contrast to the TFAAT experiment, which did not observe classification significance for the Sternberg task, this experiment consistently used a time-varying sequence of power spectral densities of size 9 for Prefrontal_β across the seven conditions. The classification potential observed in the working memory task results suggests a correlation between the prefrontal region beta band and AD risk.

**Figure 4:**
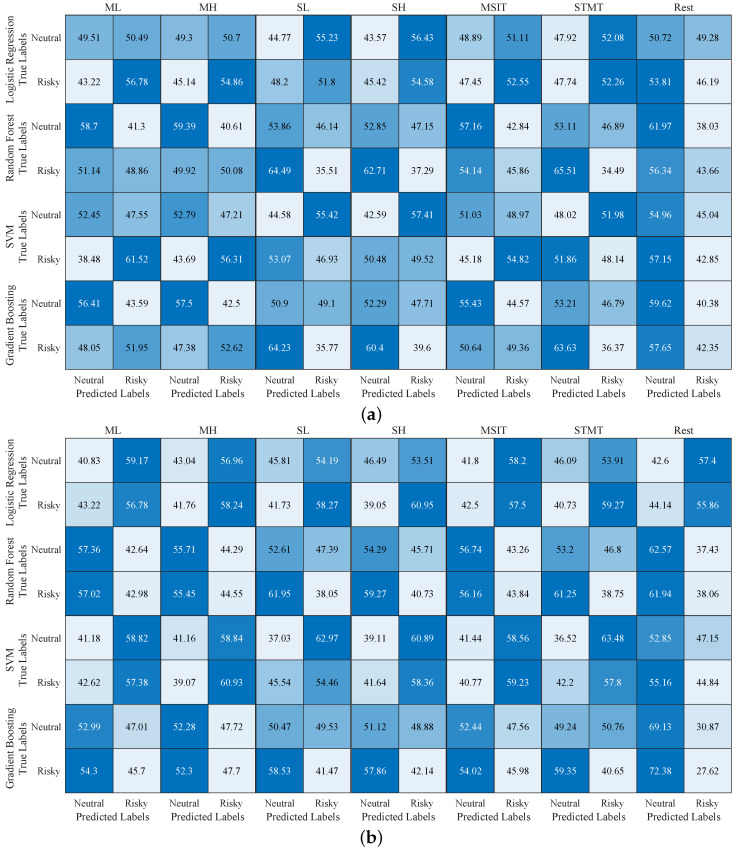
Percentage confusion matrix results of TFAAT (**a**) and PBTST (**b**) experiments. Numerical values represent percentages ranging from 0 to 100, indicating the intensity of the blue color.

The confusion matrices of the two experiments are displayed in Figure 4. It can be observed that the high classification difficulty primarily stems from the low percentage of risky classes detected in the ground truth. If the true-positive rate (TPR) of the method increases, it may lead to a higher false-positive rate (FPR), i.e., the neutral class being misclassified as risky, which is a critical error in the medical field that must be avoided. In the best case of TFAAT, ML_SVM, the TPR is 61.52%, while the FPR is 47.55%, as shown in Figure 4a. In comparing this to the other three methods, logistic regression has a lower TPR and a higher FPR, making it the least effective in classifying the classes. Meanwhile, both the Random Forest and Gradient Boosting methods exhibit low FPR but also lower TPR, leading to suboptimal classification performance. These findings are consistent with the confusion matrix results shown in Figure 2.

MSIT is similar to ML in that it achieves the best performance with the SVM method, exhibiting the highest TPR of 54.82% and a relatively low FPR of 48.97%. MH performs best with the Gradient Boosting method, showing the most balanced true-positive rate and FPR among the four methods, while the other three methods either have both rates high or both low. In the three conditions of Sternberg, the TPR was low, and the FPR was high, resulting in poor classification performance. In contrast, the Rest condition showed a detection ability only in the Random Forest method, with a TPR of 43.66% and an FPR of 38.03%.

The results of the PBTST experiment are shown in Figure 4b. For the logistic regression method, the TPR for SH is 60.95%, and the FPR is 53.51%. For SL, the corresponding values are 58.27% and 54.19%, and for STMT, they are 59.27% and 53.91%. Among these, SH achieved the best performance, with the highest TPR and a lower FPR. The remaining cases that did not show categorical significance had confusion matrices that fell into three general categories: (1) both the true-positive rate and FPR were high, (2) both were low, and (3) the FPR was higher than the TPR. These cases all resulted in nondetectability. The key to advancing future research in improving detection lies in enhancing the TPR of classification while simultaneously reducing the FPR. This dual optimization ensures that detection systems are not only sensitive to true cases but also precise in minimizing incorrect identifications, which is crucial for reliable and efficient diagnostic applications.

### 3.3. Prefrontal_β Temporal Dynamics

**Figure 5:**
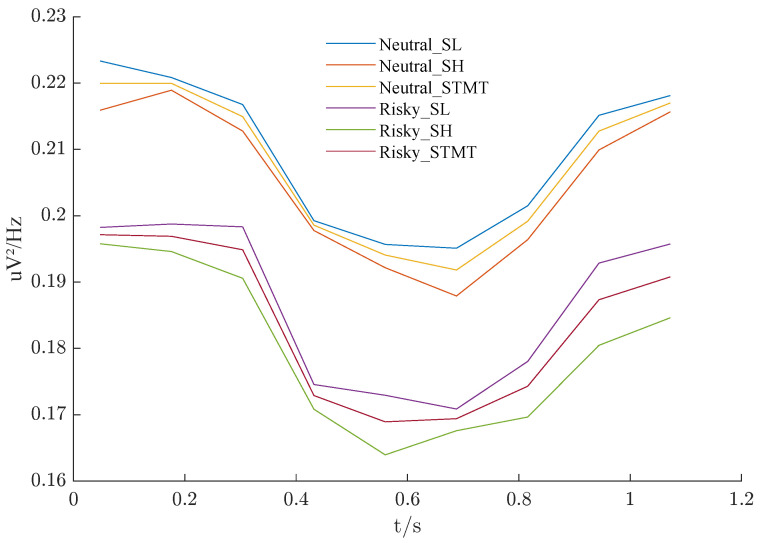
Comparison of the PSD over time for Prefrontal_β between the neutral and risky groups under the STMT and its subclasses.

In Section 3.2, we verified that the power spectral density (PSD) characteristics of the beta band in prefrontal lobe EEG exhibit certain linear separability during the Sternberg memory-type task. Based on this, we calculated the average PSD values of the neutral and risk groups for each time period and plotted the corresponding time series curves, as shown in Figure 5. It can be observed intuitively that the neutral group generally has higher PSD values than the risk group, indicating reduced high-frequency activation in AD-prone individuals. Both groups share a consistent ranking of PSD curves: SL > STMT > SH. However, the spacing between the three curves in the risk group is significantly larger than in the neutral group, resulting in the largest difference for SH. This observation aligns with the ranking in the previous section: SH > STMT > SL.

Despite amplitude differences, the overall trend of the six curves remains identical: strong beta wave activation is observed at the stimulus onset, maintained for some time, then rapidly decreases, reaching a minimum around 0.6 s before gradually increasing again. These dynamics may reveal working memory-related brain mechanisms contributing to the generation of AD risk.

## 4. Discussion

This study found that task-state features were more concentrated in the prefrontal and frontal regions. Differences in activity performance between task and resting states in the prefrontal cortex (PFC) reflect the brain’s functional regulation mechanisms under different cognitive demands [42]. As the core region for executive functions such as decision making, planning, problem solving, and inhibitory control, as well as higher cognitive functions, the PFC exhibits significant activity differences across task and resting states [43]. These findings suggest that AD risk tendencies may manifest as cognitive-related differences in EEG signals.

In all the experimental results of this paper, Rest EEG shows significant differences only in the Random Forest method of the TFAAT experiment. Random Forest is particularly suited to high-dimensional, nonlinear, and noisy data [44], demonstrating significant advantages in feature importance assessment and handling imbalanced data. The better the data features align with these characteristics, the more likely Random Forest is to perform well. Decision trees are inherently robust to noise, and the ensemble nature of Random Forest further minimizes the impact of individual noise sources on the model [45]. As EEG data are generally considered nonlinear, non-stationary, and low in signal-to-noise ratio, only the Random Forest approach achieves a detection capability with a little potential.

Only the Sternberg task combined with the logistic regression method showed significant differences in the PBTST experiment results, with SH (β-band at high task demands) being significantly higher than SL. Logistic regression excels at capturing linear relationships between features and target variables [46], modeled with log odds. When the data characteristics align with its assumptions–linear relationships, low noise, and balanced data–it often delivers comparable or even superior performance to more complex models while minimizing computational complexity and the risk of overfitting. The dorsolateral prefrontal cortex (DLPFC) is a key region of working memory, playing a vital role in goal-directed behavior and information retention [47]. The nhancement of beta-band activity in the DLPFC is typically associated with maintaining high-load task rules. Activation in the PFC has a strong correlation with working memory, as confirmed by numerous studies on Alzheimer’s disease [43]. Diminished beta-band activity in task-state EEG may reflect early cognitive deterioration, suggesting that beta-band abnormalities in middle-aged, AD-prone individuals could serve as early indicators of the disease.

Both task-state EEG analyses reveal distinct categorization abilities: MSIT demonstrates categorization capability in the TFAAT experiment, whereas STMT shows categorization ability in the PBTST experiment. This discrepancy may be attributed to the range of brain regions activated by each task. The MSIT task engages a broader range of brain regions, particularly the frontal and parietal cortices, including the anterior cingulate cortex (ACC) [48] and DLPFC, which are heavily involved in conflict modulation. Stronger activation is typically observed in tasks requiring interference suppression. In contrast, activation during the Sternberg task is relatively more localized, primarily within the PFC and parietal regions [49] directly associated with working memory. As task complexity increases, these regions may recruit additional cognitive resources to cope with the demands. Predicting AD risk in ostensibly healthy populations remains challenging due to the subtle differences in task-related EEG features. Therefore, extracting key features tailored to specific task types is critical for improving detection accuracy.

This study validates the feasibility of using EEG signals from interference inhibition and working memory states, key components of the brain’s executive functions, for predicting AD risk in healthy middle-aged adults. Although the current cross-subject classification accuracy remains low, detection capabilities are expected to improve with further research. Alternatively, for the same individuals, risk trends can be monitored over time by focusing on key features.

## 5. Conclusions

This study conducted TFAAT and PBTST experiments to explore early AD risk prediction in healthy middle-aged adults. The results demonstrate that the MSIT task achieved optimal cross-subject classification performance using the SVM approach with the TFAAT feature set, achieving a ROC AUC of 58%. Similarly, the Sternberg memory task showed classification ability using the logistic regression approach with the PBTST feature set, with a ROC AUC of approximately 54%. These findings confirm that task states possess a stronger categorization ability compared to the resting state, exhibiting significant classification potential across various scenarios. This study proposes a novel approach for early AD risk prediction in healthy populations, offering practical implications and warranting further in-depth exploration.

## Figures and Tables

**Table 1:** Distributions of the trial count available for different tasks.

Task Name	Stimulus Count	Subject Count	Trial Count (Mean ± Std)	Rate/% (Mean ± Std)	Demand Level	Trial Count (Mean ± Std)	Rate/% (Mean ± Std)
MSIT	83	62	149.69 ± 12.87	90.17 ± 7.75	Low	77.04 ± 6.68	92.82 ± 8.04
	83				High	72.65 ± 7.65	87.53 ± 9.10
STMT	72	59	116.80 ± 13.34	81.11 ± 9.26	Low	63.61 ± 6.49	88.34 ± 9.44
	72				High	53.20 ± 8.69	73.88 ± 12.60

**Table 2:** Subjects of neutral and risky groups.

Groups	ID of Subject	Count	Age	Female (%)
Neutral	1 2 6 7 8 10 13 14 15 16 17 18 19 22 23 24 25 26 28 29 31	21	55.29 ± 2.94	49.76
Risky	47 53 57 58 59 60 62 63 65 67 70 73 74 75 77 78 79 80	18	55.17 ± 3.03	51.45

**Table 3:** F24 feature subspace: results of *t*-test-based feature screening.

Tasks	Feature Subspace in F24	Total	Pre & F	Ratio
ML	Pre_δ/β, F_δ, Par_β, O_β	5	3	60
MH	Pre_δ/θ/β, F_δ, C_δ, Par_β, O_β	7	4	57.14
SL	Pre_δ/θ/β, F_δ/θ/β, T_δ α_δ, Par_δ	9	6	66.67
SH	Pre_δ/θ/α/β, F_δ/θ/β, T_δ, C_δ, Par_δ	10	7	70
MSIT	Pre_δ/θ/α/β, F_δ/θ/β, C_δ, Par_δ/β, O_δ	11	7	63.64
STMT	Pre_δ/θ/α/β, F_δ/θ/β, T_δ, C_δ, Par_δ/β, O_δ	12	7	58.33
Rest	Pre_β, F_δ/θ/β, T_θ/α, C_δ/θ/α, Par_δ/θ/α/β, O_δ/θ/α/β	17	4	23.53

Note: Pre—prefrontal; F—frontal; T—temporal; C—central; Par—parietal; O—occipital. Ratio = (Pre & F) ÷ Total × %.

**Table 4:** F216 feature subspace. Pre & F means Prefrontal and Frontal lobes.

Tasks	ML	MH	SL	SH	MSIT	STMT	Rest
Total	35	47	34	31	82	69	104
Pre & F	18	25	26	27	35	47	15
Ratio/%	51.43	53.19	76.47	87.10	42.69	68.12	14.42

## Data Availability

The preprocessed data of this study are available from the first author upon reasonable request at 2110108@stu.neu.edu.cn.

## Abbreviations

The following abbreviations are used in this manuscript:

AD	Alzheimer’s Disease;
EEG	Electroencephalography;
MSIT	Multi-Source Interference Task;
STMT	Sternberg Memory Task;
FDR	False Discovery Rate;
TFAAT	Time–Frequency Area Average Test;
PBTST	Prefrontal Beta Time Series Test;
ROC AUC	Receiver Operating Characteristic Area Under the Curve;
SVM	Support Vector Machine;
APOE	apolipoprotein E
PICALM	phosphatidylinositol binding clathrin assembly protein;
ICA	Independent Component Analysis;
ML	MSIT-Low;
MH	MSIT-High;
SL	STMT-Low;
SH	STMT-High;
DPSS	Discrete Prolate Spheroidal Sequences;
LPSO	Leave-p%-Subjects-Out;
PSD	Power Spectral Density;
PFC	Prefrontal Cortex;
DLPFC	Dorsolateral Prefrontal Cortex.
